# DMBT1 expression is down-regulated in breast cancer

**DOI:** 10.1186/1471-2407-4-46

**Published:** 2004-08-09

**Authors:** P Braidotti, PG Nuciforo, J Mollenhauer, A Poustka, C Pellegrini, A Moro, G Bulfamante, G Coggi, S Bosari, GG Pietra

**Affiliations:** 1 University of Milano, Department of Medicine, Surgery and Dentistry, S.Paolo Hospital and IRCCS Ospedale Maggiore, Milan, Italy; 2 Molecular Pathology Unit, FIRC Institute of Molecular Oncology, Milan, Italy; 3 Department of Molecular Genome Analysis, Deutsches Krebsforschungszentrum, 69120 Heidelberg, Germany; 4 Department of Pathology and Laboratory Medicine, University of Pennsylvania Medical School, Philadelphia, PA 19104, USA

## Abstract

**Background:**

We studied the expression of *DMBT1 *(deleted in malignant brain tumor 1), a putative tumor suppressor gene, in normal, proliferative, and malignant breast epithelium and its possible relation to cell cycle.

**Methods:**

Sections from 17 benign lesions and 55 carcinomas were immunostained with anti *DMBT1 *antibody (DMBTh12) and sections from 36 samples, were double-stained also with anti MCM5, one of the 6 pre-replicative complex proteins with cell proliferation-licensing functions. *DMBT1 *gene expression at mRNA level was assessed by RT-PCR in frozen tissues samples from 39 patients.

**Results:**

Normal glands and hyperplastic epithelium in benign lesions displayed a luminal polarized DMBTh12 immunoreactivity. Normal and hyperplastic epithelium adjacent to carcinomas showed a loss of polarization, with immunostaining present in basal and perinuclear cytoplasmic compartments. *DMBT1 *protein expression was down-regulated in the cancerous lesions compared to the normal and/or hyperplastic epithelium adjacent to carcinomas (3/55 positive carcinomas versus 33/42 positive normal/hyperplastic epithelia; p = 0.0001). In 72% of cases RT-PCR confirmed immunohistochemical results. Most of normal and hyperplastic mammary cells positive with DMBTh12 were also MCM5-positive.

**Conclusions:**

The redistribution and up-regulation of *DMBT1 *in normal and hyperplastic tissues flanking malignant tumours and its down-regulation in carcinomas suggests a potential role in breast cancer. Moreover, the concomitant expression of DMTB1 and MCM5 suggests its possible association with the cell-cycle regulation.

## Background

*DMBT1 *(Deleted in Malignant Brain Tumor) at chromosome 10q25.3–26.1, was considered a tumour suppressor gene because frequent deletions and/or lack of expression had been found in malignant tumours of the brain [1-3], the gastrointestinal tract [4,5], and the lung [6,7].

However, recent evidence has shown that *DMBT1 *is not a typical tumour suppressor gene because it is up-regulated in normal or inflamed epithelium adjacent to lung carcinomas as well as in some primary lung cancers and glioblastomas [8]. It was also shown that mutations in combination with loss of heterozygosity do not play a role in *DMBT1 *inactivation in many tumours including breast cancer [9,10]

*DMBT1 *is a multi-functional protein related to the Mac-2 binding protein and mucins that have a protective function and mediate cell-extracellular matrix interactions [8,11]. DMBT1^GP340^(glycoprotein-340) and DMBT1^SAG ^(Salivary Agglutinin) are *DMBT1 *variants, present in the respiratory tract and the oral cavity, respectively, that have been linked to innate host defense and epithelial regeneration [3,12-16]. In the respiratory tract, DMBT1^GP340^is involved in innate host defence by its interaction with the lectins surfactant protein D and A (Sp-D, Sp-A) and by its ability to stimulate alveolar macrophage migration [12,13,17]. Another lectin, mannan binding protein (MBP), has been shown to have an anti tumour effect against gliomas and colorectal carcinomas *in vitro *and *in vivo *[18,19]. While this anti tumor property remains to be demonstrated for Sp-A and Sp-D, we have previously reported the presence of surfactant protein A (Sp-A) in the ductal epithelium of the normal breast and its variable expression in mammary carcinomas. We found that in the normal ductal epithelium Sp-A immunoreactivity is present on the luminal side, whereas in carcinomas it is present both on the luminal side as well as on the baso-lateral surfaces. In carcinomas, the Sp-A immunoreactivity is not only redistributed but also decreased as the histological tumour grade increases [20]. Remarkably, this resembles the changes of *DMBT1 *expression and localisation observed in other tumor types originating from mono-layered epithelia such as primary esophageal adeno-carcinomas and lung carcinomas [5,8].

Hensin, the rabbit homologue of DMBT1, triggers epithelial terminal differentiation by mediating cell-extracellular matrix (ECM) interactions, and this may apply also to *DMBT1 *[3,21-24]. CRP-ductin, *DMBT1 *homologue in the mouse, and a novel homologue in the rat (*DMBT1 4,7 kb*), have been linked to initiation of cell proliferation, differentiation and repair [25,26]. It has been proposed that translocation of *DMBT1 *to the ECM triggers cellular differentiation, whereas a loss of expression of *DMBT1 *favours tumor cell growth [8].

These findings led us to explore whether similar principles apply to breast epithelium. Here we examined the expression of *DMBT1 *in the morphologically normal, hyperplastic, and neoplastic breast epithelium. We also examined in morphologically normal epithelium a possible association of *DMBT1 *expression with that of an early proliferative marker such as MCM5 (Minichromosome Maintenance protein 5). MCM5 is one of the 6 pre-replicative complex proteins (MCM 2–7) [27] and its expression was compared with that of Ki 67 proliferation index.

## Methods

### Tissue and cell-lines

From the files of the Surgical Pathology at S.Paolo University Hospital, we retrieved paraffin blocks of 72 consecutive breast lesions surgically resected. This material included 16 cases of fibrocystic disease (FD), 1 case of gynecomastia (GM), 10 samples of ductal carcinoma *in situ *(DCIS) and 45 cases of infiltrating carcinoma (IC) (36 ductal, 2 lobular, 2 mixed lobular and ductal, 2 mucinous, 1 apocrine, 1 cribriform, 1 papillary).

RT-PCR (Reverse-Transcriptase Polymerase Chain Reaction) was performed on 39 samples: i.e. 37 samples out of the 55 carcinomas used for the immunohistochemical studies, 1 sample of normal breast tissue that had been removed with a neoplastic lesion and 1 sample of fibrocystic disease. All 39 samples had been quickly frozen in liquid nitrogen after surgical resection and stored at -80°C. As confirmed by histopathologic examination, in all tumor specimens the amount of tumor cells equalled or exceeded 80% of the overall sample. As controls for each test a frozen sample of the same case positive with immunohistochemical method was used. We also analysed by RT-PCR two breast cancer cell lines (Hs578T and T-47D) obtained from the American Type Culture Collection and maintained as recommended.

### Immunohistochemistry

Sections from blocks containing the predominant lesion were incubated with anti DMBT1h12 monoclonal antibody [3] as well as anti Ki67 Mib1 (Dako Cytomation DK). Double labelling with anti-*DMBT1 *and anti MCM5 monoclonal antibodies (Novocastra Laboratories Ltd, Newcastle upon Tyne, U.K.) was performed on 17 benign lesions and a subset of 19 *in situ *and invasive carcinomas where residual normal breast tissue was well represented. On these selected samples a single incubation with anti MCM5 and anti Ki 67 antibody was performed in order to compare the two respective immunoreactivities. Antibodies were diluted as follows: anti-DMBT1h12, 1:500; anti MCM5, 1:40; anti Ki 67 Mib1, 1:100. For anti-*DMBT1 *and Ki 67 Abs, sections were pre-treated for antigen retrieval in boiling 0.01 M sodium citrate buffer pH 6, in microwave (MW) oven for 12 minutes. *DMBT1 *and MCM5 double labelling was performed in two steps following a modification of the method of Lan et al. [28], briefly: 1) first antigen retrieval 6 minutes in 0.5 M EDTA pH8 in MW oven three times, followed by 1 h incubation with anti DMBT1h12. The reaction was developed with alkaline phosphatase using Fucsin + (Dako) as chromogen. 2) same antigen retrieval followed by over-night incubation with anti MCM5 Ab and development with peroxydase and diaminobenzidine (DAB) as chromogen. All slides were incubated in an automated slides stainer (Biogenex, S.Ramon, CA, U.S.A.); reaction products were visualized by En Vision product (Dako). Negative and positive controls were added in each experiment.

Three investigators (AM, PB, PGN) evaluated separately DMBTh12 immunopositivity of morphologically normal, hyperplastic, and malignant cells. Immunopositivity was scored in 4 distinct categories according to the percentage of positive cells: 0 = negative, 1 = < 10%, 2 = 10%–50% and 3 = > 50%. MCM5 and Ki67 positivity in morphologically normal epithelium was determined with a semi-quantitative method: percentage of positive glandular structure and number of positive cells observed in each positive gland divided number of glands.

### RNA extraction

Total RNA was extracted from breast specimens using Trizol reagent, (Invitrogen s.r.l., S. Giuliano Milanese, Italy) according to the manufacturer's instructions. Total RNA was quantified and checked for purity by UV spectrophotometry.

### cDNA synthesis

Reverse transcription of RNA was done in a final volume of 20 μl containing 1x buffer (50 mM KCl, 15 mM Tris-HCl, pH 8.3), 500 μM each deoxynucleotide triphosphate, 5 mM MgCl_2_, 20 units of RNase inhibitor, 50 units of MuLV Reverse Transcriptase, 2.5 μM Random hexamers and 1 μg of total RNA. All reagents were purchased by the supplayer (Applied Biosystems, Foster City, CA, USA). The samples were incubated at 25°C for 10 min, 42°C for 60 min and at 95°C for 5 min.

### PCR amplification

Polymerase chain reaction (PCR) of the cDNA was performed in a final volume of 50 μl containing all four dNTPs (each at 200 μM), 1X buffer, 1.5 mM MgCl_2_, 1.25 units of Taq Gold DNA polymerase and each primer at 0.3 μM. The PCR amplification of *DMBT1 *was carried out in two steps, following a nested procedure. In the first step we used 2 μl of cDNA from the reverse transcription and in the second 2 μl of the first PCR product as template. Primers and thermal condition were used according to Mollenhauer et al [3]. The specificity of the PCR product was confirmed by sequencing with BigDyeTM terminator chemistry using the genetic analyser ABI Prism 310 (Applied Biosystems), β-2-microglobulin (as control for sample integrity) was amplified in the amplification mixture described above and using the following primers: 5'-TCTGGGTTTCATCCATCCGA-3' and 5'-CCCCAAATTCTAAGCAGAGTATGTAA-3'. The thermal cycling parameters were: initial step of 10 min at 94°C, 39 cycles at 94°C for 30 sec, 58°C for 30 sec, 72 °C for 30 sec, and a final step for 5 min at 72°C. All the PCR products were separated by electrophoresis on a 1.5% agarose gel containing ethidium bromide and visualized under UV transillumination. Amplification product of 602 bp (β-2-microglobulin) and 435 bp (*DMBT1*) were obtained.

### Data analysis

Statistical differences between immunopositive cases/total number of cases were calculated by Fisher's exact test. p value less than 0.05 were considered statistically significant.

T-test for independent values was used to evaluate statistically significant differences between immunoreactivity of normal and hyperplastic epithelium in benign, pre-invasive and invasive lesions.

## Results

### Immunohistochemistry

**Figure 1:**
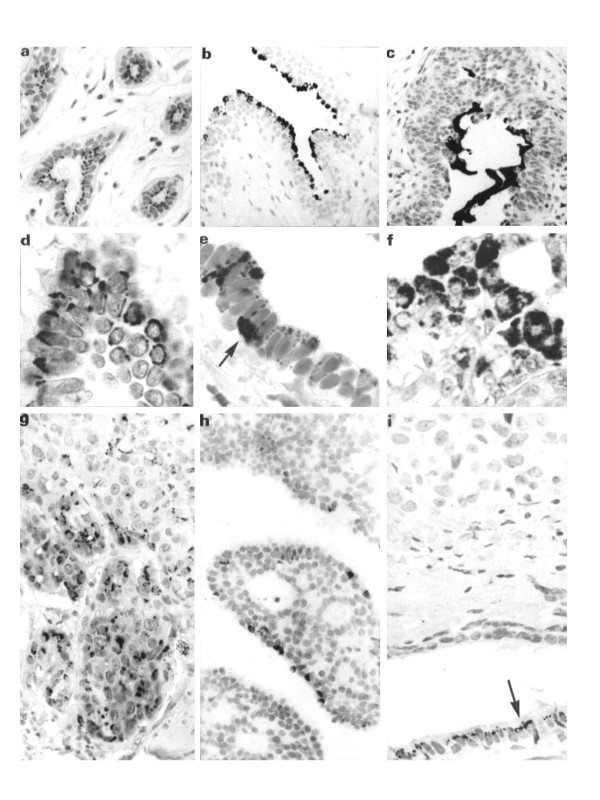
Immunoreactivity of mammary epithelium with anti DMBTh12 Ab: a, supranuclear dot positivity in morphologically normal breast sample; b, strong, abundant surface immunoreactivity in a case of fibrocystic disease; c, intraluminal immunoreactive material in a case of gynecomastia; d, numerous supranuclear and randomly distributed positive dots in intraductal papillomatosis near an infiltrating ductal carcinoma; e, strong supranuclear and baso-lateral cytoplasmic immunoreactivity of hyperplastic ductal cells in a case of mucinous infiltrating carcinoma; f, granular diffuse intracytoplasmic positivity of hyperplastic intraductal cells in a case of carcinoma *in situ*; g, numerous positive malignant tumour cells of an infiltrating ductal carcinoma; h, few positive cells in an infiltrating ductal carcinoma; i, completely negative neoplastic cells of an invasive carcinoma; the arrow points to positive cells with large elongate nuclei in a non neoplastic duct facing normal negative cells.

Different patterns of immunopositivity were observed in the histologically normal and hyperplastic breast tissue, present in the sections of benign and malignant lesions: a) single supra-nuclear, intra-cytoplasmic dot polarized toward luminal surface [Figure 1a]; b) a strong surface decoration of luminal cells [Figure 1b], c) intra-luminal immuno-reactive material [Figure 1c], d) multiple, randomly distributed peri-nuclear, irregular dots [Figure 1d], e) marked supra-nuclear and/or baso-lateral accumulation of reaction product [Figure 1e] and f) strong diffuse granular intra-cytoplasmic decoration [Figure 1f]. Morphologically normal ducts showed the pattern of immunopositivity described in section a). In addition to pattern a), the epithelium in benign lesions showed immunoreactivity patterns b) and c). Hyperplastic tissues flanking carcinoma frequently showed depolarization of immuno-reaction of patterns d), e), and f).

Normal and hyperplastic mammary structures displayed a variable DMBTh12 immunoreactivity: some ducts were decorated while others were negative. Moreover a variable percentage of cells was immunopositive. This heterogeneity suggests that there is no constitutive expression of *DMBT1 *at protein level in the mammary gland epithelium. In tumour areas, only 3 out of 55 carcinomas showed variable degrees of immunopositivity with anti-DMBTh12 Ab: in one case there was a widespread and strong decoration of numerous tumour cells (immunopositivity score = 3) with irregularly distributed dots [Figure 1g]; in the second case only few cells had cytoplasmic positivity (immunopositivity score = 1) [Figure 1h] and in the third case only weak granular decoration in a small clusters of cells was present (immunopositivity score = 1). All other carcinomas were completely negative (immunopositivity score = 0) [Figure 1i]. Down-regulation of *DMBT1 *expression in the cancerous lesions compared to the normal and/or hyperplastic epithelium was statistically significant for both, ductal carcinoma *in situ *(DCIS) (1/10 versus 33/42 normal and/or hyperplastic epithelia; p = 0.0001) and invasive carcinoma (IC) (2/45 versus 33/42 normal and/or hyperplastic epithelia; p < 0.0001).

The results of *DMBT1 *expression at protein and mRNA level are summarized in table 1.

According to the 4 category scoring system, there was no statistically significant difference of *DMBT1 *immunoreactive cells between the normal and the hyperplastic epithelium that flanked benign lesions, *in situ *and invasive carcinomas (Table 2).

**Figure 2:**
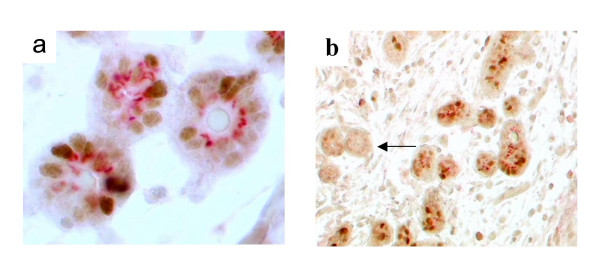
a, double staining of morphologically normal mammary epithelium: with anti DMBTh12 Ab (red stain in the cytoplasm) and with anti MCM5 Ab (Brown stain in the nucleus); b, lower magnification of double labelled mammary tissue; arrow points to a structure negative for both antigens.

**Figure 3:**
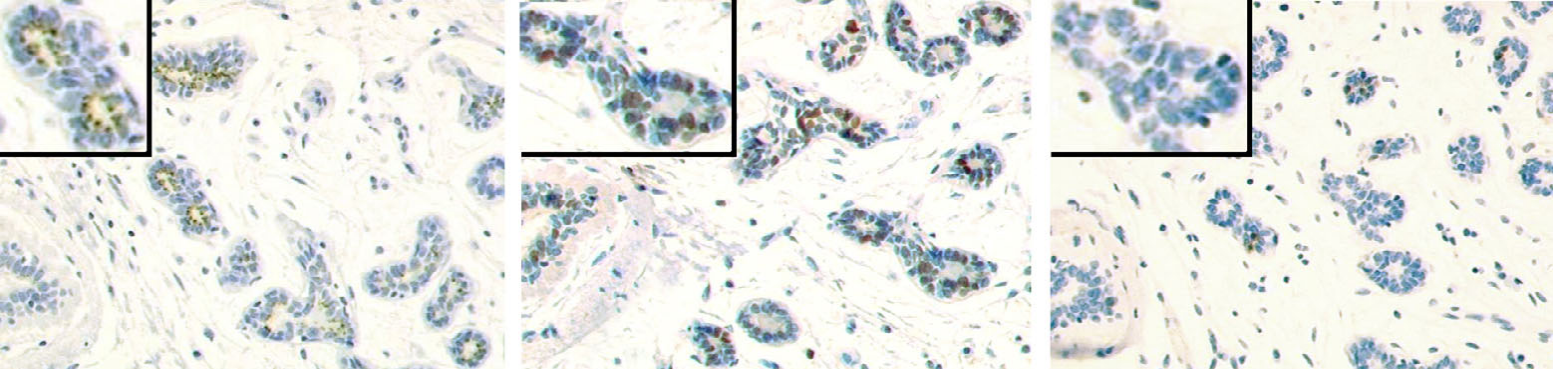
Immuno-reactivity of normal mammary epithelium in three serial sections incubated with: a, anti DMBTh12 Ab; b, anti MCM5 Ab and c, anti Ki 67 antibody where only few cells are labelled. Inset: higher magnification of the same ductal structure.

Double immunostaining with DMBTh12 Ab and anti MCM5 Ab revealed a co-expression of the two antigens, respectively, in the cytoplasm and the nucleus of the same cells in morphologically normal breast epithelium (Figure 2a,2b). Conversely, glands that were negative with anti *DMBT1 *were negative also with anti MCM5 antibody (Figure 2b). *DMBT1 *and MCM5 positive or negative glands were morphologically undistinguishable. In comparison to MCM5, very few morphologically normal epithelial cells labelled by Anti *DMBT1 *Ab, expressed Ki67 (Figure 3a,3b,3c). 30–50% of glands were positive with anti MCM5 Ab with a mean number of 4,5 positive cells per positive glandular structure versus less than 10% of positive glands with a mean number of 1,46 positive cell per positive glandular structure with anti Ki 67 Ab. As expected for a pre-replicative marker, anti MCM5 Ab decorated diffusely all benign proliferative and malignant lesions. The percentage of Ki 67 positive cells of carcinomas varied and was assessed as part of the protocol for the surgical pathology diagnosis of breast neoplasia together with other prognostic parameters i.e. hormonal receptors status and Her2 Neu positivity. These data did not correlate with *DMBT1 *expression.

### Expression analysis by RT-PCR

**Figure 4:**
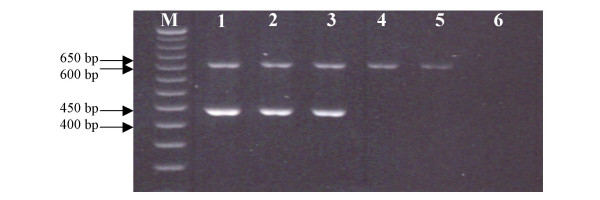
*DMBT1 *expression assessed by RT-PCR. Both *DMBT1 *(435 bp) and β-2microglobulin (602 bp) products were loaded in the same well. Lane M: size marker (50 bp ladder), lane 1: cell line Hs 578T, lanes 2, 3, 4, 5: breast carcinomas, lane 6: negative control without template.

In 28/39 cases (72%) RT-PCR confirmed immunohistochemical results, whereas 11 tumours that were negative with anti-DMBTh12 revealed PCR-products after RT-PCR amplification (Table 1). In two of these discordant cases we could document the presence in the sample of residual normal epithelial cells. *DMBT1 *mRNA was detected in both the breast carcinoma cell lines analysed: Hs 578T (Figure 4) and T47D (not shown).

The RT-PCR positivity of samples did not correlate with histological parameters or with IHC expression of hormonal receptors, proliferation index Ki 67 and Her 2/Neu.

## Discussion

In the present study we investigated *DMBT1 *expression by immunohistochemistry and RT-PCR in benign and malignant breast epithelial lesions. By immunohistochemistry we found a variable DMBTh12 positivity in the morphologically normal and hyperplastic epithelium adjacent to benign (88%) and malignant (78%) lesions. Among 55 carcinomas we could detect immunopositivity only in 3 tumours (5%); in one case of infiltrating carcinoma the immunopositivity was widespread with a pattern similar to that seen in hyperplastic lesions near carcinoma; in the other two cases (one carcinoma *in situ *and one infiltrating ductal carcinoma), it was limited to few tumour cells. *DMBT1 *expression was detected by RT-PCR in 13 of 35 (37%) infiltrating carcinomas and in 2 breast cancer cell lines. Eleven tumours, that were negative with anti-DMBTh12, revealed PCR-products after RT-PCR amplification. This discordance between IHC and RT-PCR results may be due to residual normal epithelial cells present in the sample. Furthermore other possible reasons of this discrepancy could be: 1) the greater sensitivity of molecular technique like RT-PCR in detecting very low expression levels of *DMBT1 *that were below detection by immunohistochemistry, 2) sampling problems i.e. the presence of few positive tumour cells only in the frozen samples or 3) few immunoreactive cells have been missed by microscopic examination and finally 4) *DMBT1 *protein could have an altered epitope not recognized by its paratope in the antibody. By either immunohistochemistry or molecular technique we found no correlation between *DMBT1 *expression and tumor histotype, grade, hormonal receptors status, proliferation index, Her2 Neu expression or lymph-nodal metastases.

In a recent paper on breast carcinogenesis, qualitatively similar data were reported for *DMBT1 *in human breast cancer, but an indirect weak correlation between the degree of differentiation of the breast carcinomas and the extent of the immunoreactivity was found [10]. The discrepancy in the results between our and this study may be due to sampling problems, the size of the sample and heterogeneous expression of *DMBT1 *in breast cancer or to other poorly-defined factors such as race, age or hormonal status of the population under study. Mollenhauer et al. point out that *DMBT1 *expression may be up-regulated in pathophysiological conditions such as inflammation, liver injury or after carcinogen administration and it is down-regulated in tumors to achieve a growth advantage in a dynamic and complex model. Also according to these authors, the possibility that *DMBT1 *loss may be a consequence rather than a primary event in carcinogenesis is unlikely (10).

Although the normal mammary gland has been reported to have low *DMBT1 *expression by RT-PCR [12], we found variable DMBTh12 immunopositivity in breast epithelium flanking benign and malignant lesions. Immunopositive epithelial cells in our material appeared either morphologically normal and indistinguishable from immunonegative cells or hypertrophic with abundant cytoplasm and large elongated nuclei oriented perpendicularly to the basement membrane [Figure 1i]. Moreover, DMBTh12 immunoreactivity was also maintained in hyperplastic epithelium flanking carcinomas. This resembles the *DMBT1 *expression patterns observed in the human respiratory tract and in associated inflammation and carcinomas [8] and suggest that the variable *DMBT1 *expression in the normal mammary gland epithelium may result from the necessity of *DMBT1 *induction. Indeed, in parallel *in vivo *studies, it was found that *DMBT1 *is strongly up-regulated in the mammary gland tissue of the mouse shortly after carcinogen-administration and prior to the onset of histo-pathologically visible symptoms [10]. It has been reported that Sp-D and Sp-A are ligands of *DMBT1 *products [29]. We recently demonstrated that Sp-A, in breast carcinoma tissues, has a pattern of expression resembling that of its interaction partner [20]; thus, we hypothesize that co-ordinated induction of both SP-A and *DMBT1 *in breast tissue may be part of a protective response as suggested by Kang et al [30] for *DMBT1 *and Sp-D in pulmonary epithelium.

While this interaction may be attributed to lumenally secreted *DMBT1*, the depolarisation of the staining patterns indicates that *DMBT1 *may also be secreted to other destinations, i.e. the ECM, by the mammary gland epithelial cells. In this regard, the second putative function of *DMBT1*, i.e. triggering of epithelial differentiation, is noteworthy. It may prevent hyperproliferative epithelial cells from out-of control proliferation and progression to the tumor state. This view is supported by two present findings. The correlation between MCM5 and *DMBT1 *expression may point to a coupling to cell-cycle processes: basolateral relocation and hyper-expression of *DMBT1 *at the ECM may provide cells that are licensed to proliferate with protection from unregulated proliferation. On the other hand loss of *DMBT1 *expression in a significant numbers of DCISs and ICs is consistent with the notion that *DMBT1 *inactivation may offer growth advantages to neoplastic cells [10]. The loss of *DMBT1 *expression appears to take place early, i.e. already at the time of transition from hyperplastic lesions to carcinoma, as it has been reported in oesophageal squamous cell carcinomas [5].

The exact mechanisms for *DMBT1 *inactivation remain unclear. An inactivation by mutation appears not to be a major mechanism for *DMBT1 *inactivation in tumors [10,31] The studies of Munoz and co-workers suggest that epigenetic silencing of either *DMBT1 *or genes that regulate *DMBT1 *expression takes place in some tumors [32]. Accordingly, except for direct epigenetic silencing of the *DMBT1 *promotor, loss of expression or mutation of *DMBT1*-regulatory genes might represent alternative candidate mechanisms.

## Conclusions

We have shown that *DMBT1 *expression and cellular location in breast epithelium varies in normal, hyperplastic and neoplastic cells. In normal or hyperplastic tissue flanking carcinomas it is up-regulated and polarized toward glandular lumen or the basolateral membrane while it is down-regulated in carcinomas. These findings closely resemble the patterns observed for its interaction partner SP-A in an earlier study. Additionally we have shown that *DMBT1 *is co-expressed with MCM5 in normal and hyperplastic tissue. We hypothesize that hyper-expression and basolateral relocation of *DMBT1 *in these epithelia prevents the out of control growth that characterizes neoplastic epithelium.

## List of abbreviations used

DMBT1: Deleted in Malignant Brain Tumor 1,

MCM5: Minichromosome Maintenance protein, 5

RT-PCR: Reverse Transcriptase Polymerase Chain Reaction

GP 340: Glycoprotein 340

SAG: salivary Agglutinin

Sp-A, Sp-D: Surfactant Protein A, D

MBP: Mannan Binding Protein

ECM: Extracellular Matrix

FD: Fibrocystic Disease

GM: gynecomastia

DCIS: Ductal Carcinoma *In Situ *

IC: Invasive Carcinoma

MW: Micro Wave

EDTA Ethylenediaminetetraacetic Acid

DAB: Diaminobenzidine

## Competing interests

None declared.

## Autors Contributions

PB designed the study, participated in the evaluation of immunohistochemical results and drafted the manuscript. PGN participated in the evaluation of immunohistochemical results and performed statistical analysis. JM participated in sequence alignment, criticism and produced DMBTh12 antibody. AP participated in sequence alignment and data evaluation. CP Carried out RT-PCR. AM participated in the evaluation of histologic samples and immunohistochemical results. GB participated in data evaluation and sequence alignment GC participated in study design and data evaluation. SB participated in study design, sequence alignment, criticism and coordinated molecular study. GGP participated in study design, criticism and general revision of paper.

## Pre-publication history

The pre-publication history for this paper can be accessed here:



## Figures and Tables

**Table 1:** Immunohistochemical and RT-PCR expression of *DMBT1 *in benign and malignant mammary epithelium as number of positive cases/total number of cases. We considered positive the cases in which we found any percentage of positive cells.

	FD/GM	Normal/Hyperplastic	DCIS	IC
IHC	15/17 (88%)	33/42 (78%)	1/10 (10%)	2/45 (4%)
RT-PCR	1/1	1/1	0/2	13/35 (37%)

IHC= immunohistochemestry FD/GM = fibrocystic disease and gynecomastia Normal/Hyperplastic = morphologically normal and/or hyperplastic tissues flanking carcinoma DCIS = ductal carcinoma *in situ *IC = invasive carcinoma

**Table 2:** DMBTh12 staining scores in normal (N) and hyperplastic epithelium (H) flanking fibrocystic disease and gynecomastia (FD/GM), ductal carcinoma *in situ *(DCIS) and invasive carcinoma (IC)

Immunohistochemical postivity score	Epithelium type	FD/GM cases/total number*	DCIS cases/total number*	IC cases/total number*
0	N	4/14	0/8	11/37
	H	3/14	0/4	16/22
1	N	0/14	6/8	16/37
	H	1/14	1/4	10/22
2	N	8/14	1/8	9/37
	H	8/14	2/4	4/22
3	N	2/14	1/8	1/37
	H	2/14	1/4	2/22

* total number of cases in which was present normal or hyperplastic epithelium.

## References

[B1] Mollenhauer J, Wiemann S, Scheurlen W, Korn B, Hayashi Y, Wilgenbus KK, Von Deimling A, Poustka A (1997). DMBT1, a new member of the SRCR superfamily, on chromosome 10q25.3–26.1 is deleted in malignant brain tumors. Nat Genet.

[B2] Somerville RP, Shoshan Y, Eng C, Barnett G, Miller D, Cowell JK (1998). Molecular analysis of two putative tumor suppressor genes, PTEN and DMBT, which have been implicated in glioblastoma multiforme disease progression. Oncogene.

[B3] Mollenhauer J, Herbetz S, Holmskov U, Tolnay M, Krebs I, Merlo A, Schroder HD, Maier D, Breitling F, Wiemann S, Grone HJ, Poustka A (2000). *DMBT1 *Encodes a Protein Involved in the Immune Defense and in Epithelial Differentiation and is Highly Unstable in Cancer. Cancer Res.

[B4] Mori M, Shiraishi T, Tanaka S, Yamagata M, Mafune K, Tanaka Y, Ueo H, Barnard GF, Sugimachi K (1999). Lack of *DMBT1 *expression in oesophageal, gastric and colon cancers. Brit J Cancer.

[B5] Mollenhauer J, Herbertz S, Helmke B, Kollender G, Krebs I, Madsen J, Holmskov U, Sorger K, Schmitt L, Wiemann S, Otto HF, Gröne HJ, Poustka A (2001). Deleted in Malignant Brain Tumors 1 Is a Versatile Mucin-like Molecule Likely to Play a Differential Role in Digestive Tract Cancer. Cancer Res.

[B6] Takeshita H, Sato M, Shiwaku HO, Semba S, Sakurada A, Hoshi M, Hayashi Y, Tagawa Y, Ayabe H, Horii A (1999). Expression of the *DMBT1 *gene is frequently suppressed in human lung cancer. Jpn J Cancer Res.

[B7] Wu W, Kemp BL, Proctor ML, Gazdar AF, Minna JD, Hong WK, Mao L (1999). Expression of DMBT1, a Candidate Tumor Suppressor Gene, Is frequently Lost in Lung Cancer. Cancer Res.

[B8] Mollenhauer J, Helmke B, Müller H, Kollender G, Lyer S, Diedrichs L, Holmoskov U, Ligtenberg T, Herbetz S, Krebs I, Wiemann S, Madsen J, Bikker F, Schmitt L, Otto HF, Poustka A (2002). Sequential changes of the *DMBT1 *expression and location in normal lung tissue and lung carcinomas. Genes Chromosomes Cancer.

[B9] Mollenhauer J, Müller H, Kollender G, Lyer S, Diedrichs L, Helmke B, Holmoskov U, Ligtenberg T, Herbetz S, Krebs I, Madsen J, Bikker F, Schmitt L, Wiemann S, Scheurlen W, Otto HF, von Deimling A, Poustka A (2002). The SRCR/SID region of *DMBT1 *defines a complex multi-allele system representing the major basis for its variability in cancer. Genes Chromosomes Cancer.

[B10] Mollenhauer J, Helmke B, Medina D, Bergmann G, Gassler N, Müller H, Lyer S, Diedrichs L, Renner M, Wittig R, Blaich S, Hamann U, Madsen J, Holmskov U, Bikker F, Ligtenberg A, Carlén A, Olsson J, Otto HF, O'Malley B, Poustka A (2004). Carcinogen Inducibility in Vivo and Down-Regulation of *DMBT1 *During Breast Carcinogenesis. Genes Chromosomes Cancer.

[B11] Mollenhauer J, Deichmann M, Helmke B, Muller H, Kollender G, Holmskov U, Ligstemberg T, Krebs I, Wiemann S, Bantel-Shaal U, Madsen J, Bikker F, Klauk SM, Otto HF, Moldenhauer G, Pouskta A (2003). Frequent downregulation of *DMBT1 *and galectin-3 in epithelial skin cancer. Int J Cancer.

[B12] Holmskov U, Mollenhauer J, Madsen J, Vitved L, Grønlund J, Tornøe I, Kliem A, Reid KBM, Poustka A, Skjødt K (1999). Cloning of gp-340, a putative opsonin receptor for lung surfactant protein D. Proc Natl Acad Sci USA.

[B13] Tino MJ, Wright JR (1999). Glycoprotein-340 Binds Surfactant Protein-A (SP-A) and Stimulates Alveolar Macrophage Migration in an SP-A-Independent Manner. Am J Resp Cell Mol.

[B14] Prakobphol A, Xu F, Hoang VM, Larsson T, Bergstrom J, Johansson I, Frängsmyr L, Holmskov U, Leffler H, Nilsson C, Borén T, Wright JR, Strömberg N, Fisher SJ (2000). Salivary Agglutinin, Which Binds *Streptococcus mutans *and *Helicobacter pylori*, Is the Lung Scavenger Receptor Cysteine-rich Protein gp-340. J Biol Chem.

[B15] Ligtenberg TJ, Bikker FJ, Groenink J, Tornoe I, Leth-Larsen R, Veerman EC, Nieuw Amerongen AV, Holmskov U (2001). Human salivary agglutinin binds to lung surfactant protein-D and is identical with scavenger receptor protein gp-340. Biochem J.

[B16] Kang W, Reid KBM (2003). DMBT1, a regulator of mucosal homeostasis through the linking of defense and regeneration?. FEBS Letters.

[B17] Holmskov U, Lawson P, Teisner B, Tornøe I, Willis AC, Morgan C, Koch C, Reid KBM (1997). Isolation and Characterization of a New Member of the Scavenger Receptor Superfamily, Glycoprotein-340 (gp-340), as a Lung Surfactant Protein-D Binding Molecule. J Biol Chem.

[B18] Fujita T, Taira S, Kodama N, Matsushita M, Fujita T (1995). Mannose-binding protein recognizes glioma cells: in vitro analysis of complement activation on glioma cells via the lectin pathway. Jpn J Cancer Res.

[B19] Ma Y, Uemura K, Oka S, Kozutsumi Y, Kawasaki N, Kawasaki T (1999). Antitumor activity of mannan-binding protein *in vivo *as revealed by a virus expression system: Mannan-binding protein dependent cell-mediated cytotoxicity. Proc Natl Acad Sci USA.

[B20] Braidotti P, Cigala C, Graziani D, Del Curto B, Dessy E, Coggi G, Bosari S, Pietra GG (2001). Surfactant Protein A Expression in Human Normal and Neoplastic Breast Epithelium. Am J Clin Pathol.

[B21] Takito J, Hikita C, Al-Awqati Q (1996). Hensin, a New Collecting Duct Protein Involved in the In Vitro Plasticity of Intercalated Cell Polarity. J Clin Invest.

[B22] Al-Awqati Q, Vijayakumar S, Hikita C, Chen J, Takito J (1998). Phenotipic plasticity in the intercalated cell: the hensin pathway. Am J Physiol.

[B23] Vijayakumar S, Takito J, Hikita C, Al-Awqati Q (1999). Hensin Remodels the Apical Cytoskeleton and Induces Columnarization of Intercalated Epithelial Cells: Processes that Resemble Terminal Differentiation. J Cell Biol.

[B24] Hikita C, Vijayakumar S, Takito J, Erdjument-Bromage H, Tempst P, Al-Awqati Q (2000). Induction of Terminal Differentiation in Epithelial Cells Requires Polymerization of Hensin by Galectin 3. J Cell Biol.

[B25] Cheng H, Bjerknes M, Chen H (1996). CRP-ductin: A Gene Expressed in Intestinal Crypts and in Pancreatic and Hepatic Ducts. Anat Rec.

[B26] Bisgaard HC, Holmskov U, Santoni-Rugiu E, Nagy P, Nielsen O, Ott P, Age E, Dalhoff K, Rasmussen LJ, Tygstrup N (2002). Heterogeneity of Ductular Reactions in Adult Rat and Human Liver Revealed by Novel Expression of Deleted in Malignant Brain Tumor 1. Am J Pathol.

[B27] Stoeber K, Tlsty TD, Happerfield L, Thomas GA, Romanov S, Bobrow L, Williams ED, Williams GH (2001). DNA replication licensing and human cell proliferation. J Cell Sci.

[B28] Lan HY, Mu W, Nikolic-Paterson DJ, Atkins RC (1995). A novel, simple, reliable, and sensitive Method for multiple immunoanzyme staining: use of microwave oven heating to block antibody crossreactivity and retrieve antigens. J Histochem Cytochem.

[B29] Crouch E, Wright JR (2001). Surfactant proteins A and D and pulmonary host defense. Annu Rev Physiol.

[B30] Kang W, Nielsen O, Fenger C, Madsen J, Hansen S, Tornoe I, Eggleton P, Reid KBM, Holmskov U (2002). The scavenger receptor, cysteine-rich domain-containing molecule gp-340 is differentially regulated in epithelial cell lines by phorbol ester. Clin Exp Immunol.

[B31] Mueller W, Mollenhauer J, Stockhammer F, Poustka A, von Deimling A (2002). Rare mutations of the *DMBT1 *gene in human astrocytic gliomas. Oncogene.

[B32] Munoz J, Lazcoz P, del Mar Inda M, Nistal M, Pestana A, Encio IJ, Castresana JS (2004). Homozygous deletion and expression of PTEN and *DMBT1 *in human primary neuroblastoma and cell lines. Int J Cancer.

